# Integrative Conjugative Element ICE*Hs*1 Encodes for Antimicrobial Resistance and Metal Tolerance in *Histophilus somni*

**DOI:** 10.3389/fvets.2018.00153

**Published:** 2018-07-10

**Authors:** Krishna Bhatt, Edouard Timsit, Neil Rawlyk, Andrew Potter, Karen Liljebjelke

**Affiliations:** ^1^Faculty of Veterinary Medicine, University of Calgary, Calgary, AB, Canada; ^2^Vaccine and Infectious Disease Organization-International Vaccine Centre, University of Saskatchewan, Saskatoon, SK, Canada

**Keywords:** *Histophilus somni*, antimicrobial resistance, metal tolerance, copper, zinc, integrative conjugative element, feedlot cattle

## Abstract

The objectives of this study were to determine antimicrobial resistance and metal tolerance, and identify associated genes and mobile genetic elements in clinical strains of *Histophilus somni* isolated from feedlot cattle in Alberta during years 2012–2016 (contemporary isolates, *n* = 63) and years 1980–1990 (historical isolates, *n* = 31). Comparison of antimicrobial resistance (AMR) showed a significant increase in resistance among contemporary isolates compared to historical isolates (*P* < 0.001). Tolerance to copper (Cu) and zinc (Zn) concentrations above 1 mM was observed in 68 and 52% of the contemporary isolates, respectively. The *tet*(H) gene associated with oxytetracycline resistance and multicopper oxidase (*mco*) and cation efflux (*czcD*) genes associated with Cu and Zn tolerance were identified. An integrative conjugative element; ICE*Hs1*, was identified in whole genome sequences of strains resistant to oxytetracycline, which had Cu and Zn minimum inhibitory concentrations (MIC) >1 mM. The length of ICE*Hs1* was 64,932 bp and it contained 83 genes, including tetracycline resistance gene *tet*H, a multidrug efflux pump gene *ebrB*, and metal tolerance genes *mco, czcD*, and *acr3*. Comparative genomics of ICEs revealed that ICE*Hs1* shares high homology with previously described ICEs of *Histophilus somni, Pasteurella multocida*, and *Mannheimia haemolytica*. The ICE*Hs1* is an active element capable of intra- and inter-genus transfer as demonstrated by successful transfer to *H. somni* and *P. multocida* recipients. All isolates carrying ICE*Hs1* were resistant to tetracycline, a commonly used antibiotic in feedlots, and had Cu and Zn MIC higher than 1 mM. Since Cu and Zn are routinely used in feedlots, there is the possibility of co-selection of AMR in *H. somni* due to selection pressure created by Cu and Zn. Based on results of *in-vitro* conjugation experiments, ICE*Hs1* mediated transmission of antimicrobial and metal resistance genes is possible between BRD pathogens in the respiratory tract, potentially undermining treatment options available for histophilosis and BRD.

## Introduction

*Histophilus somni* causes a multisystemic disease condition known as histophilosis, which is an important cause of mortality in feedlot cattle in western Canada, with mortality rates up to 2% of the herd (1). Histophilosis can manifest as myocarditis, pericarditis, bronchopneumonia, pleuritis, polyarthritis, thrombotic meningoencephalitis (TME), conjunctivitis, or otitis (2). Histophilus can be associated with 50–60% of the morbidity in feedlot cattle (3). Economic losses associated with histophilosis occur through mortality, cost of medication, low average daily gain and decreased carcass quality. The ability of *H. somni* to infect multiple organs creates challenges in its prevention and control. Although various strategies, including vaccination and mass medication with antimicrobials (metaphylaxis) are used for disease control, histophilosis still remains a significant cause of disease in fall placed calves in western Canada (4).

Antimicrobials are used in feedlots for treatment and prevention of diseases and for improved feed efficiency. The respiratory form of histophilosis occurs as a component of Bovine Respiratory Disease (BRD), and protocols in feedlots are designed to control both histophilosis and BRD (3, 4). Antimicrobials used for treatment and control of histophilosis and BRD in Canada are: oxytetracycline, tilmicosin, tulathromycin, florfenicol, enrofloxacin, and ceftiofur (5). More recently, gamithromycin, and tildipirosin were labeled for treatment and control of BRD in Canada (5). Antimicrobials in use to enhance feed efficiency are chlortetracycline, chlortetracycline-sulfamethazine, and ionophores. Tylosin is used for prevention and control of liver abscesses (6, 7).

Use of antimicrobials in cattle feedlot facilities creates selection pressure in bacteria, leading to increased prevalence of antimicrobial resistance (8). A field trial conducted using long-acting oxytetracycline for metaphylaxis in auction market-derived calves in Canada did not detect a significant reduction in the risk of *H. somni* mortalities, and the authors hypothesized one reason to be antimicrobial resistance (4). Multiple studies conducted in Canada and the USA have shown a gradual decrease in susceptibility of *H. somni* to antimicrobials commonly used in feedlots (9, 10). There is no official surveillance system in Canada for monitoring antimicrobial resistance in respiratory pathogens of cattle. Changes in antimicrobial use may have altered the susceptibility of *H. somni* to common antimicrobials. It is therefore important to monitor antimicrobial susceptibility to ensure effective treatment and control of histophilosis.

Copper (Cu) and zinc (Zn) are regularly used in the diet of feedlot cattle as part of multi-mineral supplements (11). The National Research Council (NRC) recommendation for Cu and Zn inclusion in beef cattle feed is 10 and 30 mg/kg of rations, respectively (11). However, a survey of feedlot nutritionists reported that the actual inclusion of Cu and Zn is 1.8 and 3.1 times higher than NRC recommendations (12). In Alberta, supplementation of Cu and Zn up to 15 and 100 mg/kg of diet, respectively, are used for arriving feedlot calves (13). Although Cu and Zn are important for a variety of cellular and biochemical processes, in higher concentrations they are cytotoxic (14). Copper and Zn toxicity in bacteria is due to redox potential, interaction with thiol groups (R-SH), and competition with other metal ions for metal binding sites in proteins (14). To counter toxicity, bacteria living in metal-rich environments may acquire metal tolerance genes through horizontal DNA transfer (15).

There are several reports describing linkage between antibiotic resistance and metal tolerance in bacteria isolated from food animals (16, 17). Involvement of mobile genetic elements explains the linkage between metal tolerance and antibiotic resistance, because they can carry both antibiotic and metal resistance genes and they play an important role in the horizontal transmission of resistance. Integrative conjugative elements (ICE) are a type of mobile genetic element which are self-transmissible and carry machinery for their excision, transfer, and integration (18). Integrative conjugative elements carrying various antimicrobial resistance genes have been identified among members of the *Pasteurellaceae* family (19–22). The ICE identified in *P. multocida* 36950; ICE*Pmu1*, and in *Mannheimia haemolytica* M42548; ICE*Mh1* has been characterized (19, 21). The whole-genome sequence of *H. somni* 2336 includes an ICE designated as ICE*Hso*2336 (22). The transferability of ICE*Hso*2336 has not been demonstrated. In this study we identified an ICE in *H. somni* strains from Alberta, characterized phenotype and genotype for metal and antimicrobial resistance, and determined its ability to transfer horizontally.

The aim of this study was to (1) determine and compare antimicrobial, copper, and zinc resistance in *H. somni* isolates collected during 2012–2016 (contemporary isolates) and 1980–1990) (historical isolates); (2) identify and characterize ICE, and ICE-associated antimicrobial and metal resistance genes.

## Materials and methods

### Bacterial culture, isolation, and identification

*H. somni* isolates were collected from necropsy tissue samples of feedlot cattle in Alberta that died or were euthanized displaying clinical signs of histophilosis or BRD in the 1980's (*n* = 31), which we refer to as “historical isolates,” and during 2012–2016 (*n* = 63), which we refer to as “contemporary isolates.” The tissue samples from which the historical isolates were isolated were heart, lung, synovial fluid, pleural swab, and brain (only from TME suspects). The historical isolates were obtained from the repository of Vaccine and Infectious Disease Organization-International Vaccine Center (VIDO-InterVac), Saskatoon, SK, Canada. Isolation and identification of contemporary isolates were performed as described previously by Madampage et al. (23). Tissues from which contemporary isolates were isolated were heart, lung, and joint fluid. Some contemporary isolates were isolated from deep nasal swabs. The tissue samples were homogenized in 1 mL of Brain Heart Infusion Broth (Hardy Diagnostics, CA, USA) and plated directly onto Trypticase Soy Agar Plates (Hardy Diagnostics, CA, USA) with 5 % defibrinated sheep blood (Dalynn Biologicals, AB, Canada). Plates were incubated at 37°C in the presence of 5% CO_2_ for 48 h. Colonies showing characteristics of *H. somni* were re-streaked onto Columbia Blood Agar plates and incubated as above. A colony PCR assay was performed to amplify the 16s ribosomal RNA gene specific to *H. somni* for identification. The primer pair used was; forward (5′-GAAGGCGATTAGTTTAAGAG-3′) and reverse (5′-TTCGGGCACCAAGTATTCA-3′) (23). *H. somni* ATCC 700025 strain and *E. coli* ATCC 25922 strains were used as positive and negative controls, respectively. Isolates confirmed as *H. somni* were stored on cryogenic beads (Prolab Diagnostics, ON, Canada) at −80°C.

### Antimicrobial susceptibility assay

The Sensititre® system (Trek Diagnostic Systems, Cleveland, OH, USA) and commercially available bovine/porcine antimicrobial plates containing 18 different antimicrobials in two-fold dilutions was used for antimicrobial susceptibility testing. The inoculated plates were read after 18–24 h of incubation at 35°C in 5% CO_2_ using the Opti-Read^TM^ system (Trek Diagnostic Systems, Cleveland, OH, USA). Based on minimum inhibitory concentrations (MIC) obtained, susceptibility was determined using CLSI established breakpoints. The breakpoints established in earlier studies were used when assaying neomycin and tylosin susceptibility (24, 25).

### Copper and zinc tolerance assay

Because there was no established protocol for metal susceptibility testing in *H. somni*, we created an agar dilution assay for assaying Cu and Zn tolerance. Copper Sulfate (CuSO_4_.5H_2_O) and Zinc Sulfate (ZnSO_4_.7H_2_O) (Alfa Aesar, Massachusetts, USA), were used to make pH-adjusted Muller-Hinton chocolate agar medium plates containing various concentrations of Cu and Zn in a two-fold dilution series. Muller-Hinton chocolate agar plates without added salts were prepared as controls. To show that the antibacterial effect was due to the cations Cu^++^ or Zn^++^ rather than the anion ${\text{SO}}_{4}^{--}$, agar plates containing sodium sulfate (Na_2_SO_4_) (Alfa Aesar, Massachusetts, USA) in the same concentration series were also prepared.

A bacterial inoculum was prepared in 5 mL of cation-adjusted Muller-Hinton broth (CAMHB) and a metal replicator was used for inoculation. Agar plates were incubated at 35°C in the presence of 5% CO_2_ for 18–24 h and were read manually. Three replications for each experiment using fresh agar plates and inoculum were performed to assess reproducibility of the metal tolerance assay. Because there are no established breakpoints for Cu and Zn for *H. somni*, the MIC values obtained were compared with the pan-susceptible reference strain (*H. somni* ATCC 700025) for analysis.

### Polymerase chain reaction (PCR)

A PCR assay was conducted on isolates exhibiting phenotypic resistance to detect metal and antimicrobial resistance genes. Genomic DNA was used as template, extracted using the boiling method and PrepMan® Ultra Sample Preparation Reagent (Applied Biosystems Inc., ON. Canada). A Taq PCR Core Kit (Qiagen Inc., ON, Canada) was used to prepare the master mix for PCR reactions. Each PCR reaction (25 μL) in final concentration contained 1X PCR buffer with 1.5 mM of MgCl_2_, 200 μM of each dNTP, 0.4 μM of each primer (forward and reverse) and 1.25 units of Taq polymerase. Amplification was performed in a T100™ Thermal Cycler (Bio-Rad, CA, USA) with the following conditions: Initial denaturation at 94°C for 3 min; 30 cycles of: denaturation at 94°C for 30 s, annealing at 50–65°C (depending upon primer) for 30 s, and extension at 72°C for 1 min; with a final extension at 72°C for 3 min. Primer pairs used in assays screening isolates for specific genes were designed by the authors using the whole genome sequence of isolate AVI 1. The primer pairs used, target genes, predicted amplicon sizes, and annealing temperatures are shown in Table 1. Agarose gel electrophoresis was performed after PCR assay to visualize presence of targeted amplicons of expected molecular size. The PCR amplicons were purified, sequenced, and sequences were used to confirm gene identity using the BLAST algorithm.

**Table 1:** Primer pairs used for screening metal and antimicrobial resistance genes and ICE-associated genes in *H. somni* strains.

**PCR target**	**Resistance phenotype**	**PCR primer sequences (5′-3′)-F/R**	**Annealing temp (°C)**	**Predicted amplicon size (bp)**	**Primer reference**
*tet*(H)	OXY	ATACTGCTGATCACCGT/ TCCCAATAAGCGACGCT	60	1076	D'Amours et al. 26
*ebrB*	MDR	CACCTGCCAAAATCAAGCCAA/ CGAAGTGTTTGGCTCAACGA	54	254	This work
*mco*	Cu	TGGCCTAATGCCAACTGAGG/ GTTCCTTGCGTTTCACCCAC	56	813	This work
*mco type 3*	Cu	CCGTGATCCATTCCCTGCAT/ AGGGCGATACGGTGGAAATC	56	844	This work
*HEP*	Cu	TCGGGGGTGCTGTCTTTTAC/ GTCGGCGTGTATTTTGAGGC	56	1136	This work
*czcD*	Zn	TAATTCCGCCCAAGCCCTTT/ ATCGGGTTCAACTTCGCTGT	54	475	This work
**ICE-ASSOCIATED GENES**					
Integrase		CGGAATCATAGACCTGCCACT/ TGCAGTTGTATGTCGGAATCG	58	729	This work
Transposase		CCTGTTTCAATGCCAAAGTTTCCT/ GCTCCCTTCAACACCACAAG	58	170	This work
SSD		TGTATAACGCTCTTGCCCGT/ GGCAACGATCCTGAAATGCG	54	252	This work

*bp, base pairs; OXY, oxytetracycline; MDR, Multidrug resistance; mco, multicopper oxidase; HEP, Heavy metal efflux pump; SSD, Single-stranded DNA binding protein*.

### Whole genome sequencing

Genomic DNA for whole genome sequencing was extracted using QIAamp® genomic DNA kit following manufacturer's instructions (Qiagen, Toronto, ON, Canada) (23). Whole genome sequencing of three isolates (strains AVI 1, AVI 14, and AVI 31) was performed using Illumina Miseq (Illumina, San Diego, California, USA) with paired-end 150 base pair read type at Cofactor Genomics (Saint Louis, Missouri, USA) (23, 27). These isolates were selected for whole genome sequencing based on their phenotypic characteristics. Two strains, AVI 1 and AVI 31, were resistant to oxytetracycline and had copper and zinc MIC ≥ 1 mM, while strain AVI 14 was susceptible to oxytetracycline and had copper and zinc MIC ≤ 1 mM. These isolates were collected from cases of myocarditis in different feedlots.

### Genome assembly, annotation, and identification of ICE

The raw paired-end read sequences of each isolate of *H. somni* provided by Cofactor genomics in FASTQ format were converted into FASTA format. The software tool Geneious 6.1.7 (http://www.geneious.com) was used for *de novo* assemblies of the genome (23, 27). The contigs were assembled from the FASTA reads. A draft whole genome of each isolate was created based on assembled contigs. Gene prediction and annotation was performed using Prokka software (https://www.vicbioinformatics.com/software.prokka.shtml). For genes in the ICE which were rendered as hypothetical proteins by Prokka, a homolog search was conducted using BLASTx and BLASTp against the non-redundant nucleotide database (http://www.ncbi.nlm.nih.gov/blast/Blast.cgi), and the annotation was then manually curated using Artemis. The open reading frames designated as hypothetical proteins were also searched for specific functions against the Inter-Pro databases (https://www.ebi.ac.uk/interpro/) and TIGRFAM protein database (http://www.jcvi.org/cgi-bin/tigrfams/index.cgi). Amino acid alignments for putative proteins with hits below 40% identity or alignment less than 120 base pairs were discarded (28).

Identification of ICE in the isolates was completed by pairwise alignment of the putative ICE*Hso*2336 to the assembled genome of the isolates. Comparison of ICE*Hs1* with ICE*Pmu1*, ICE*Mh1*, ICE*Hso*2336 was performed using Easyfig; a genome comparison visualizer (29).

### Conjugation assay

Conjugation assay was performed to examine mobility of ICE*Hs1* between *H. somni* isolates, between *H. somni* and *P. multocida*, and between *H. somni* and *E. coli*. The plasmid-free *H. somni* strains AVI 1 and AVI 31, which carry ICE*Hs1*, served as donor strains. A rifampicin resistant (Rif^r^) *H. somni* strain devoid of ICE as demonstrated by whole genome sequencing and susceptible to tetracycline, Cu and Zn, was used as recipient strain. The rifampicin-resistant *P. multocida* DSM 16031 and sodium azide-resistant *E. coli* J53 strain were used as recipients to assess host range of ICE*Hs1*. Spontaneous rifampicin resistant mutants of recipient strains were generated by selecting on Columbia Blood Agar (CBA) plates containing increasing concentrations (1, 5, 10, 25, 50, 100, 150, 200 μg/mL) of rifampicin (Alfa Aesar, Massachusetts, USA).

Overnight cultures of donor and recipient strains in BHITT were used for mating experiments as described in other studies (21, 30). In brief, one mL broth culture of donor and recipient was centrifuged at 13,000 RPM for a minute, and the cell pellet was washed with 500 μL of 0.85% sodium chloride followed by suspension in 30 μL of BHITT broth. A 1:1 mixture of donor and recipient was spotted onto CBA and incubated at 37°C in the presence of 5% CO_2_ for 24 h. Colonies from mating spots were collected in 1 mL of BHITT broth, serially diluted, and plated on the appropriate selection agar plates. The colony forming units (CFU) of transconjugants and recipients were counted after an incubation period of 48 h, and transfer frequency was calculated as number of transconjugants per recipient cell. Representative colonies of transconjugants were re-streaked twice onto appropriate selection plates for purification. The identity of streak-purified transconjugants was confirmed using the Sensititre® system. Susceptibility assays were used to confirm transfer of Cu and Zn tolerance and antimicrobial resistance. The presence of metal and antimicrobial resistant genes along with ICE*Hs1*- associated genes were confirmed by PCR assay. For the PCR assay of resistance genes, original donor, and recipient were used as a positive and negative controls, respectively.

### Statistical analysis

*Histophilus somni* isolates were grouped as susceptible or resistant per outcome of antimicrobial susceptibility assays. The resistant category included both intermediate and resistant isolates. The percentage of resistant contemporary and historical isolates were calculated and compared using Fisher's exact test. The Mann-Whitney U test was used to compare the MIC of contemporary and historical isolates for antimicrobial, Cu, and Zn susceptibility assays.

The association between metal tolerance and antimicrobial resistance was analyzed by multivariable logistic regression in SPSS version 21.0. To facilitate logistic regression analysis, results of metal tolerance tests were categorized into two groups; high tolerance and low tolerance. The high tolerance group consisted of *H. somni* isolates with MIC value >1 mM, and the low tolerance group had MIC value less than or equal to 1 mM. Metal tolerance was taken as an outcome variable (Dichotomous variable). Resistance to various antimicrobials (resistant/susceptible) were taken as independent variables. A univariable logistic regression analysis was performed to identify all the possible independent variables. The independent variables associated with metal tolerance with *P* < 0.2 were considered for initial inclusion in the multivariable logistic regression analysis. The collinearity among all the eligible independent variables was tested using the Spearman rank correlation coefficient. The collinearity was considered to be present if the correlation coefficient was >0.70. In the case of collinear variables, separate models were run and the best model was selected based on Akaike Information Criteria (AIC). The models were built manually using a backward elimination process and the independent variables with *P* > 0.05 were excluded from the final model. The remaining variables in the model were accessed for confounding by detecting >30% changes in the coefficients with the removal of a variable. The goodness-of-fit of the final model was evaluated using Hosmer-Lemeshow Chi-square test. Separate models were run for Cu and Zn tolerance. Due to lack of precision, convergence, and statistical stability of the model, antimicrobials having less than 2% resistance were not evaluated.

## Results

### Antimicrobial susceptibility

**Figure 1:**
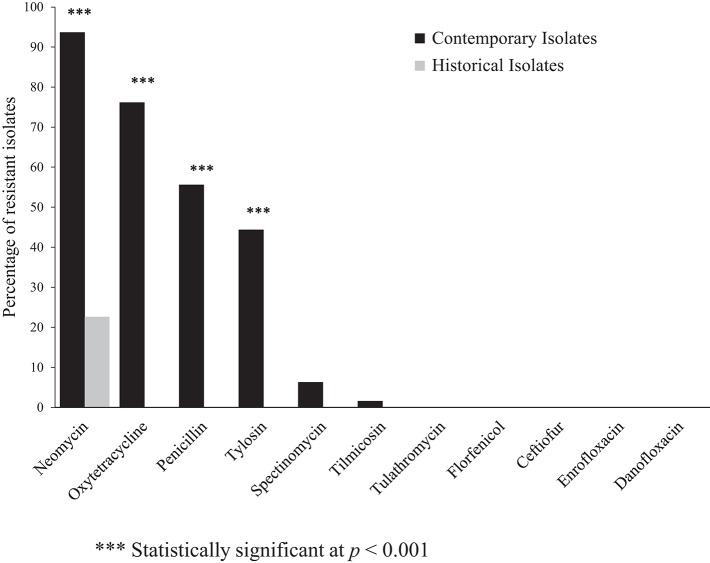
Percentage of antimicrobial resistant contemporary (2012–2016), (*n* = 63) and historical (1980's), (*n* = 31) clinical isolates of *Histohilus somni* from feedlot cattle in Alberta. Fisher's exact test was used for statistical comparison of antimicrobial resistance between contemporary and historical isolates.

The percentage of resistance to a panel of antimicrobials among the contemporary and historical isolates is shown in Figure 1. The contemporary isolates were most frequently resistant to neomycin (93.6%), oxytetracycline (76.2%), penicillin (55.6%), and tylosin (44.4%). The historical isolates (*n* = 31), were susceptible to all antimicrobials assayed, except neomycin, against which 22% of the isolates demonstrated resistance. The differences in the frequency of resistance against neomycin, oxytetracycline, penicillin, and tylosin between the historical and contemporary isolates was highly statistically significant at *P* < 0.001 (Figure 1).

Multidrug-resistance, here defined as resistance to ≥3 antimicrobial classes, was detected in 62% of the contemporary isolates; however, multidrug-resistance was not identified among the historical isolates. The most frequently observed multidrug-resistance pattern was: aminoglycosides-tetracyclines-β-lactams. Multidrug-resistant isolates were most frequently resistant to penicillin within the β-lactam class, and neomycin among the aminoglycoside class of antimicrobials.

In addition to the increased frequency of both single-drug and multi-drug resistance among the contemporary isolates as compared to the historical isolates, a statistically significant (*P* ≤ 0.001) increase in MIC values for penicillin, gentamicin, neomycin, tylosin, oxytetracycline, and chlortetracycline was identified using the Mann-Whitney U test. A 128 fold increase in the MIC for oxytetracycline was observed. The median MIC value for contemporary and historical isolates and calculated fold-increase in median MIC is shown in Table 2.

**Table 2:** Changes in MIC_50_ of various antimicrobials for contemporary strains (2012–2016), (*n* = 63) compared to historical (1980's), (*n* = 31) strains of *Histophilus somni* isolated from feedlot cattle in Alberta.

**Antimicrobials**	**Median MIC value (MIC**_**50**_**)**			
	**Historical**	**Contemporary**	**Fold Increase^a^**	***P*** **value^b^**
Ceftiofur	0.25	0.25	1	1
Danofloxacin	0.125	0.125	1	0.1
Enrofloxacin	0.125	0.125	1	1
Ampicillin	0.25	0.25	1	0.08
Penicillin	0.125	0.25	2	<0.001
Clindamycin	0.5	0.5	1	0.1
Gentamicin	8	16	2	<0.001
Spectinomycin	16	16	1	0.06
Neomycin	16	32	2	<0.001
Tulathromycin	8	4	0.5	0.2
Tilmicosin	1	8	8	0.001
Tylosin	4	8	2	<0.001
Florfenicol	0.25	0.5	2	1
Oxytetracycline	0.125	16	128	<0.001
Chlortetracycline	0.5	1	2	<0.001
Tiamulin	2	2	1	0.6

a *Calculated by dividing median MIC value of contemporary isolates by historical isolates. One-fold increase indicates no change in MIC*.

b *Mann-Whitney U test was used for statistical comparison*.

### Copper and zinc susceptibility

**Figure 2:**
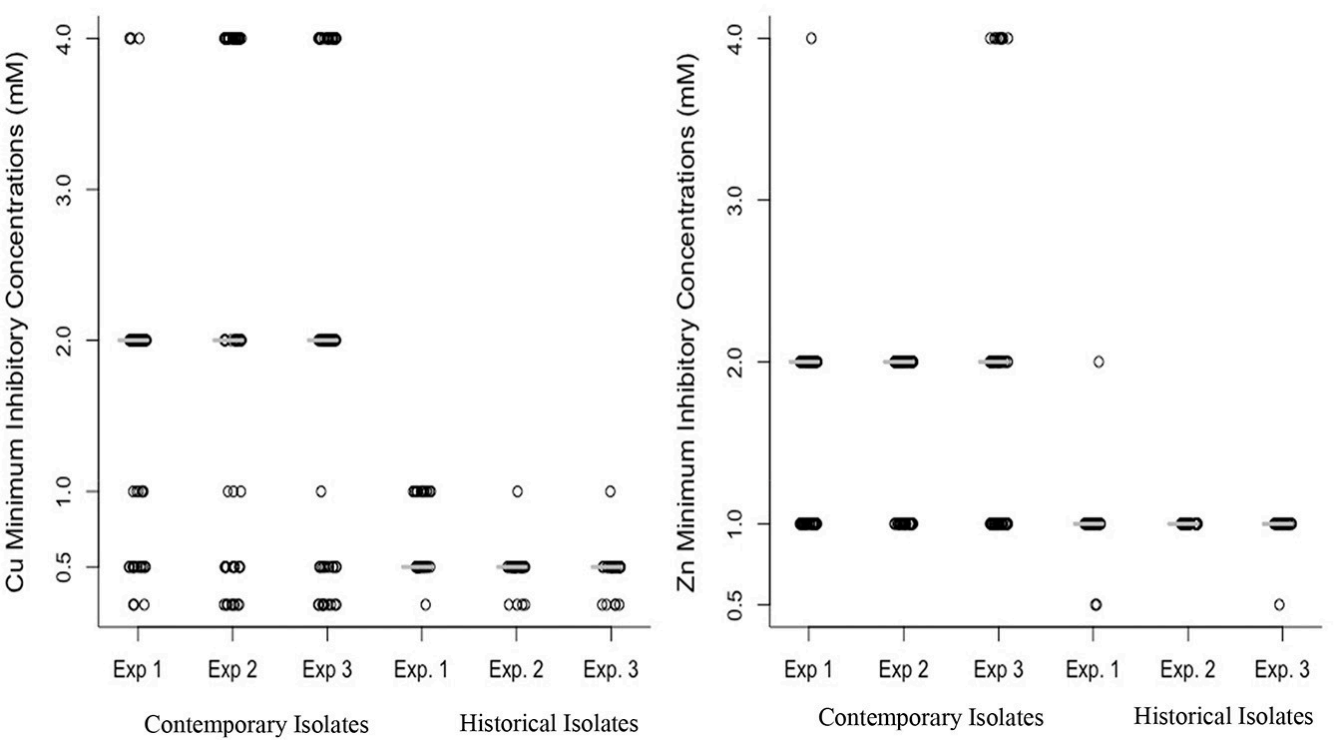
Distribution of minimum inhibitory concentration (mM) of copper (left) and zinc (right) for 63 contemporary (2012–2016) and 31 historical (1980's) isolates of *Histophilus somni*. The MIC value for the isolates tested was in triplicate in three replicate experiments (Experiments 1 and 2). The median value of copper and zinc in contemporary and historical isolates was consistent in all three experiments, indicating the reproducibility of the metal tolerance assay. A Mann-Whitney U test was used for comparing median MIC between historical and contemporary isolates and a significant difference was detected (*P* < 0.001).

The results of three replicate Cu and Zn tolerance assays are shown in Figure 2. The median MIC for Cu for contemporary isolates was significantly higher (2 mM) than the median MIC for the historical isolates (0.5 mM), as determined using the Mann-Whitney U test (*P* < 0.001). All of the historical isolates had an MIC value ≤ 1 mM, with a large proportion (87%) of isolates clustering at MIC 0.5 mM (Figure 2). Among the contemporary isolates, the MIC values for Cu ranged from 0.25 to 4 mM. Among the contemporary isolates there appears to be a bimodal distribution of MIC values, with one-third (33%) of isolates clustering at 0.5 mM and 1.0 mM concentrations, and two-thirds (68%) of isolates clustering at MIC values 2.0 mM and 4.0 mM.

The median MIC for Zn among contemporary isolates was also significantly higher (2 mM) when compared to the MIC for historical isolates (1 mM), as determined using the Mann-Whitney U test (*P* < 0.001). All of the historical isolates had Zn MIC values equal to or lower than 1 mM, with a large proportion (97%) clustering at 1 mM concentration (Figure 2). Among the contemporary isolates, the MIC values for Cu ranged from 0.5 to 4 mM. Among the contemporary isolates 52% had an MIC higher than 1 mM. Figure 2 illustrates the increase in the median MIC for both Zn and Cu among the contemporary isolates as compared to the historical isolates.

### Association between copper, zinc, and antimicrobial resistance

Logistic regression analysis found a statistically significant (*P* ≤ 0.05) association between metal tolerance and antimicrobial resistance phenotypes among the contemporary isolates of *H. somni*. In the univariable analysis, resistance to either oxytetracycline or zinc (MIC > 1.0 mM) were significantly associated with resistance to copper (MIC > 1 mM) (*P* ≤ 0.01), as shown in Table 3. The final model shows a sufficient fit with the included predictors (Hosmer-Lemeshow Chi-square test, *P* = 0.26). The odds of an isolate having copper MIC >1 mM was 37 times higher in oxytetracycline resistant strains compared to susceptible strains (OR = 37, *P* = 0.002) (Table 4). *Histophilus somni* isolates having a zinc MIC >1 mM were 44 times more likely to have a copper MIC >1 mM (OR = 44.8, *P* < 0.004). The univariable analysis for zinc tolerance revealed that resistance to oxytetracycline, tylosin, penicillin, and Cu were all significantly associated with having a zinc MIC >1 mM (*P* ≤ 0.01) (Table 3). In multivariable analysis, only the variable of having copper MIC >1 mM remained significantly associated with having zinc MIC >1 mM. (OR = 54, 95% confidence interval, 6.4–452.6, *P* < 0.001). Multidrug-resistance phenotypes was significantly associated with having an MIC >1.0 mM for both copper and zinc.

**Table 3:** Univariable analysis of association between metal tolerance and antimicrobial resistance in clinical isolates of *H. somni* from feedlot cattle in Alberta (*n* = 63).

**Metal tolerance (MIC > 1 mM)**	**Resistant phenotype^a^**	**Odds ratio**	**95% Confidence interval**	***P* value**
Copper	Oxytetracycline	45.5	8.1–253.3	<0.001
	Tylosin	4.9	1.4–17.4	0.01
	Neomycin	7.9	0.8–83.3	0.08
	Zinc	54.0	6.4–452.6	<0.001
	Multidrug resistance	14.5	3.9–54.8	<0.001
Zinc	Oxytetracycline	13	2.4–64.7	0.002
	Penicillin	14.7	4.3–49.9	<0.001
	Neomycin	3.8	0.4–38.8	0.19
	Tylosin	2.4	0.9–6.7	0.09
	Copper	54.0	6.4–452.6	<0.001
	Multidrug resistance	7.6	2.4–24.3	0.001

a *Corresponding susceptible category was taken as reference category for calculating odds ratio*.

**Table 4:** Multivariable analysis of association between copper tolerance and antimicrobial resistance in clinical isolates of *H. somni* from feedlot cattle in Alberta (*n* = 63).

**Resistant phenotype^a^**	**Odds ratio**	**95% Confidence interval**	***P* value**
Oxytetracycline	37.0	3.6–380.8	0.002
Zinc	44.8	3.5–575.3	0.004

a *Corresponding susceptible category was taken as reference category for calculating odds ratio*.

### Identification of genes responsible for metal tolerance

A subset of contemporary isolates having either or both copper or zinc MIC above 1 mM, in which we had previously identified the *tet*(H) gene (*n* = 32) by PCR, were screened by PCR assay for the heavy metal tolerance genes multicopper oxidase *mco*, multicopper oxidase type 3 (*mco* type 3), heavy metal efflux pump *czcD*, and heavy metal efflux pump HEP. Among this subset of contemporary isolates, 68% (23/34) were positive for the multi-copper oxidase *mco*, and 88% (30/34) were positive for *czcD*. All of the isolates screened by PCR assay for these metal tolerance genes were negative for the *mco* type 3 and heavy metal efflux pump HEP gene.

### Structure of ICE*Hs1* and comparative genomics

An integrated conjugative element was detected by genomic analysis of assembled genome sequences of *H. somni* AVI 1 and AVI 31 strains. This ICE was designated as ICE*Hs1* following the nomenclature proposed by Burrus et al. (31), which suggests using the ICE followed by the initials of the organism in italics and the strain number. The GenBank accession number for the nucleotide sequence is: MF136609.1 (https://www.ncbi.nlm.nih.gov/genbank).

**Figure 3:**
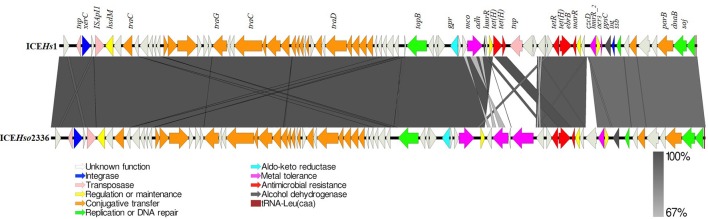
Schematic structure of ICE*Hs*1 identified in this study compared to previously described ICE*Hso*2336. The 83 open reading frames (ORFs) present in the ICE*Hs*1 are represented by arrows with the arrowhead representing direction of transcription. The ORFs are color coded according to their biological function. The area between the ICEs shaded in dark gray indicates 100% and light gray indicates 67% shared sequence similarity based on the BLASTn. Function of genes present in ICE region have been inferred from BLAST hits and annotation with PROKKA.

**Figure 4:**
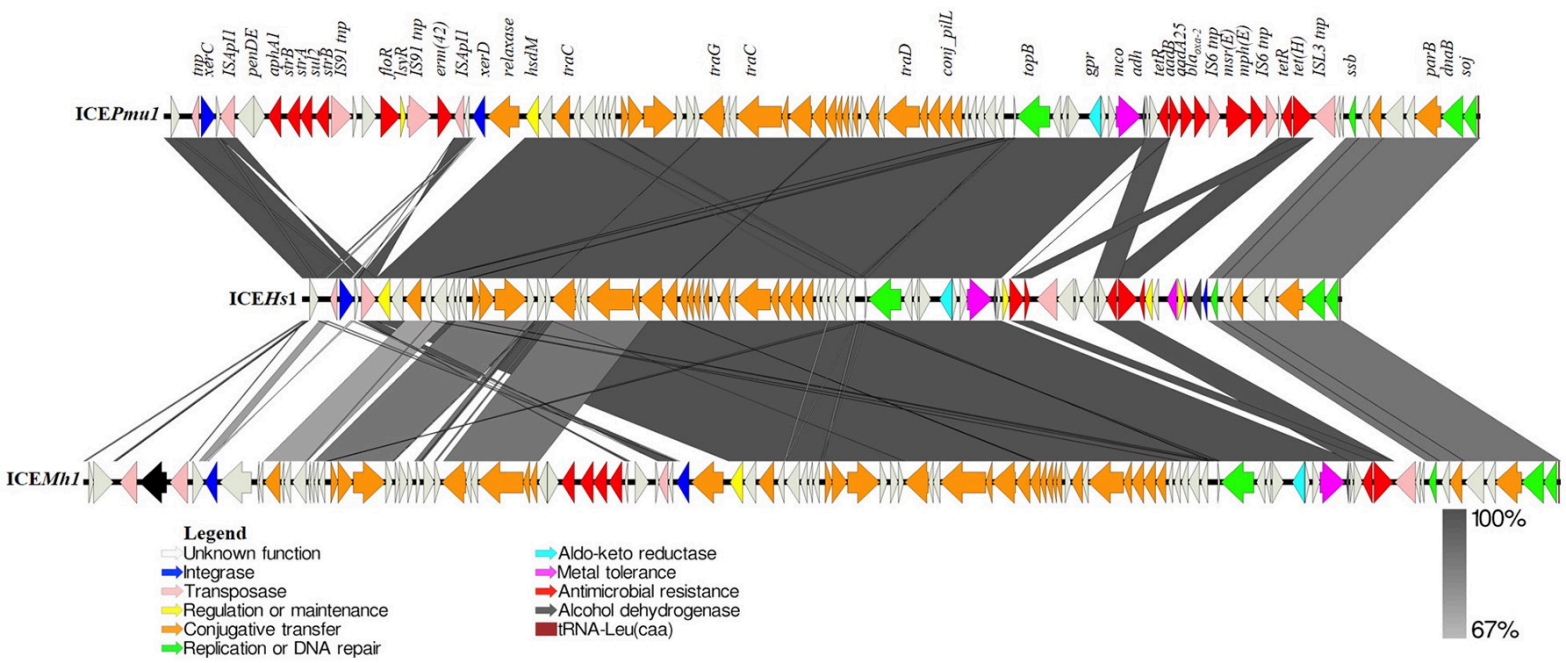
Schematic comparison of ICE*Hs*1 identified in this study with ICE*Pmu1* and ICE*Mh1*. Open reading frames (ORFs) present in each ICE are represented by arrows with the arrowhead representing direction of transcription. The ORFs are color coded according to their biological function. The areas between the ICEs shaded in dark gray indicates 100% and light gray indicates 67% shared sequence similarity based on the BLASTn. Function of genes present in ICE region have been inferred from BLAST hits and annotation with PROKKA. These three ICEs from three common BRD pathogens (*P. multocida, H. somni*, and *M. haemolytica*) share 90% nucleotide identity. ICE*Pmu1* and ICE*Mh1* carry 12 and 5 antimicrobial resistance genes, respectively. However, ICE*Hs*1 carries only *tet*(H) and a multidrug efflux pump gene (*ebrB*), along with 3 different metal tolerance genes, among which only two were present in ICE*Mh1* and ICE*Pmu1*.

The length of ICE*Hs1* was 64,932 bp and the sequence contains 83 open reading frames. The GC content of ICE*Hs1* was 39.7%, which is only slightly higher than the GC content of the entire genome of the *H. somni* AVI 1 strain (37.4%). Sequence comparison shows that ICEHs1 shares ~100% nucleotide sequence identity with the previously described ICEHso2336 up to base 44,142. This region is indicated by the dark gray bar in Figure 3. The high degree of sequence homology indicates this is a highly conserved region of the ICE. After base 44,142, ICEHs1 shares only ≥67% sequence identity with ICEHso2336. This variable region is indicated by a light gray bar in Figure 4. Overall pairwise sequence identity between the two ICEs is 91.7% (Figure 3).

Comparison of the sequences shows that both ICEHs1 and ICEHso2336 are integrated at the tRNA^Leu^ site in their respective genomes and are flanked by the characteristic 11 bp (GATTTGAATC) direct repeats and have an 86-bp tRNALeu at the right terminus. ICE*Hs1* was integrated into the fourth of the four copies of tRNA^Leu^ in the *H. somni* AVI 1 and 13 strains.

Sequence analysis of ICEHs1 identified three copies of the tetracycline resistance gene *tet*H, a multidrug efflux pump gene *ebrB*, the metal tolerance genes *mco, czcD*, and the arsenic resistance gene *acr3*. ICEHs1 did not contain the mco type 3 and heavy metal efflux pump hep genes, which are present in ICE*Hso*2336 (Figure 3).

Sequence comparison between ICE*Hs*1 in *H. somni* AVI, ICE*Pmu*1 in *P. multocida* 36950, ICE*Mh*1 in *M. haemolytica* M42548 is shown in Figure 4. Comparative genomics of these ICEs indicates that ICE*Hs1* shares high identity with the ICEs of BRD pathogens *P. multocida* and *M. haemolytica* (Figure 4). The ICE's ICE*Mh1* and ICE*Pmu1* were 92345 bp and 82214 bp long and contained 107 and 88 genes, respectively. The length of ICE*Hs1* is shorter at 64932 bp, and the sequence contains 83 genes. The comparison of the three ICEs in Figure 4 show a mosaic structure consisting of conserved and variable regions. The conserved regions share more than 99% homology between ICE*Hs*1, ICE*Pmu*1, and ICE*Mh*1.

### Conjugal transfer of ICE*Hs*1

In a conjugation assay, *H. somni* donor strains AV1 1 and AVI 31 transferred ICE*Hs1* to pan-sensitive *H. somni* strains at a frequency of 10–7 per recipient (Table 5). ICE*Hs1* was transferred at a frequency of 10–8 per recipient to a pan-sensitive strain of *P. multocida* (Table 5). Susceptibility testing of the recipient strains pre- and post-conjugation demonstrated that the MIC for tetracycline, Cu, and Zn increased to the same values as those of the donor strain in both species of recipients (Rifr H. somni AVI 14 and Rifr P. multocida DSM 16031). The transfer of ICE*Hs*1 was confirmed using PCR assay to detect the relevant ICE-associated resistance genes (Table 5). The mating experiments performed between *H. somni* donor strains and the recipient strain *E. coli* J53 were not successful, and these matings were not pursued further.

**Table 5:** Transfer frequency of ICE*Hs1*, and phenotypic and genotypic characteristics of ICE*Hs1* donor (AVI 1 and AVI 31 strains), recipient (Rif^r^ *H. somni* AVI 14 and Rif^r^ *P. multocida* DSM 16031) and transconjugant strains.

**Strains**	**ICE*Hs1* transfer frequency**	**Phenotype MIC (**μ**g/mL)**			**Genotype**					
		**tet**	**Cu**	**Zn**	***tet*(H)**	***ebrB***	***mco***	***czcD***	**int**	**tnp**
*H. somni* AVI 1	Donor	16	4	2	+	+	+	+	+	+
*H. somni* AVI 31	Donor	16	2	2	+	+	+	+	+	+
*H. somni* AVI 14 Rif^r^	Recipient	0.25	0.25	1	–	–	–	–	–	–
*P. multocida* DSM 16031 Rif^r^	Recipient	1	2	1	–	–	–	–	–	–
*H. somni* AVI 1 → AVI 14 Rif^r^	1.16 × 10^−7^	16	4	2	+	+	+	+	+	+
*H. somni* AVI 31 → AVI 14 Rif^r^	1.23 × 10^−7^	16	2	2	+	+	+	+	+	+
*H. somni* AVI 1 → *P. multocida* DSM 16031 Rif^r^	1.57 × 10^−8^	16	4	2	+	+	+	+	+	+

*Rif^r^, rifampicin resistant; tet, tetracycline; Cu, Copper; Zn, Zinc; tet(H), tetracycline resistance gene; ebrB, small multidrug efflux transporter gene; mco, Multicopper oxidase gene; czcD, cation efflux transporter gene; int, Integrase; tnp, transposase*.

## Discussion

The resistance phenotypes for the contemporary isolates of *H. somni* used in this study are similar to those reported for *H. somni* collected from diseased cattle in southern Alberta in 2016 (32). The frequency of oxytetracycline resistance in our contemporary *H. somni* strains (76%), is similar that from a recent study by D'Amours et al. (26). Resistance to oxytetracycline among BRD pathogens isolated from beef cattle might be a result of the historically extensive use of tetracyclines in feedlots. Long-acting oxytetracycline is commonly used in western Canada for metaphylactic treatment to prevent BRD and histophilosis (4, 33). Feed-grade oxytetracycline, chlortetracycline, and chlortetracycline-sulfamethazine are also used in feedlot rations for the control and prevention of histophilosis (3, 26, 34).

We identified the *tet*(H) gene in 91.7% of the oxytetracycline-resistant isolates in this study. This finding is in agreement with previous studies which found the *tet*(H) gene to be common in *H. somni* from feeder cattle in Alberta (30, 26). The *tet*(H) gene has also been identified in *P. multocida* and *M. haemolytica*, along with the *tet*(B) and *tet*(G) genes (21, 35).

The majority of beef cattle feedlots in North America use tylosin in feed rations to prevent and control liver abscesses (6). In addition, an injectable form of tylosin tartrate is used in Alberta for prevention of implant site abscesses (34). In spite of frequent use, resistance to tylosin has rarely been reported in cattle pathogens (25). The resistance observed in some of the contemporary *H. somni* isolates in this study could theoretically be associated with its use in feedlot rations.

It is surprising that more than 90% of our isolates are neomycin resistant, as there have been few reports of neomycin resistance in BRD pathogens. Although some commercial neomycin-oxytetracycline products are labeled for feed efficiency and enteric diseases in beef cattle, use of this formulation is infrequent. The *ebrB* gene detected in our neomycin-resistant isolates might be responsible for the neomycin resistant phenotype observed, because *ebrB* encodes an MDR efflux pump of the small multidrug resistance protein (SMR) family of efflux pumps, which mediate resistance to narrow spectrum beta-lactams (penicillin) and aminoglycosides (neomycin), in addition to quaternary ammonium compounds (36, 37).

Multidrug-resistance (resistance to ≥3 antimicrobial classes) was detected in 62% of the contemporary isolates; however, multi-drug resistance (MDR) was absent among the historical isolates. The most frequently observed MDR pattern was aminoglycosides-tetracyclines-β-lactams. Isolates were most frequently resistant to penicillin within the β-lactams and neomycin among the aminoglycosides, which, again may be explained by the carriage of an MDR efflux pump. The detection of MDR in these isolates could pose a problem for the feedlot industry due to the potential for decreased treatment options and increased chance of treatment failure.

A significantly higher percentage of strains resistant to oxytetracycline, penicillin, or neomycin was observed among the contemporary isolates (*P* < 0.001) as compared to the historical isolates. In addition to the increase in percentage of resistance, there was a significant increase in median MIC values for the contemporary isolates as compared to the historical isolates (Mann-Whitney U test, *P* ≤ 0.001). The 128-fold fold-increase in median MIC for oxytetracycline among the contemporary isolates as compared to the historical isolates is a clinically significant finding.

The *H. somni* isolates in this study were collected from cattle that were euthanized or died manifesting clinical signs of BRD or histophilosis. It is possible these isolates may have been exposed to antimicrobials when the animals were treated for disease. A higher prevalence of AMR is generally reported in isolates collected from diseased cattle compared to isolates collected from healthy cattle (24), therefore the prevalence of resistance in our isolates should be interpreted with caution, as these data may overestimate resistance among *H. somni* in healthy cattle (32, 38).

The development of metal tolerance in bacteria residing in metal-rich environments such as mining sites and industrial areas is well documented (39). However, the explanation for acquisition of metal tolerance in cattle pathogens such as *H. somni* is not completely understood. It can be hypothesized that tolerance to metals must provide survival advantage to *H. somni* in competitive environments. Copper and zinc are added in higher concentration than physiological need, to feedlot rations (12, 40). A large proportion of Cu and Zn fed to cattle is excreted in urine and manure (15), and not surprisingly, a significant amount of Cu (32.3–730.1 mg/kg), and Zn (75.9–4333.8 mg/kg) can be detected in cattle manure and agricultural soil near feedlot operations (41). *Histophilus somni* is transmitted either by ingestion of infectious agents from nasal or urogenital secretions or by inhalation of aerosolized droplets (3, 42). It is possible that *H. somni* may be exposed to high concentrations of these metals in the environment, feed bunks, water, pen-floor, manure, or urine. Although the actual bacterial load present in feed, water, pen-floor, or feedlot dust is not known, it has been shown that *H. somni* can survive in these environments in nasal secretions for more than 2 months (43). In such context, having Cu and Zn tolerance might provide a competitive advantage for environmental survival.

To the best of our knowledge, this is the first study assaying the MIC for Cu and Zn in *H. somni*. The MIC of the reference strain (*H. somni* ATCC 700025) was 1 mM for both Cu and Zn, and this value was used for interpretation of results. The rationale for using the ATCC *H. somni* strain as a control is that the strain was pan-susceptible to antimicrobials, decreasing the possibility that the control strain carries MDR efflux pumps which may contribute to metal tolerance and antimicrobial resistance. While the MIC of the control strain was used to discriminate the isolates as having high tolerance or low tolerance to Cu and Zn, we cannot classify strains as resistant or susceptible, only more or less tolerant. The differences in tolerance of the *H. somni* isolates to various concentrations of zinc and copper above the 1 mM threshold implies there are various genetic mechanisms contributing to the phenotype. The metal tolerance assay clearly demonstrated an increase in the median MIC for both zinc and copper among the contemporary isolates compared to the historical isolates.

The statistical association observed between metal tolerance and antimicrobial resistance phenotypes is supported by detection of antimicrobial resistance genes physically linked with metal tolerance genes in the newly described Integrated Conjugative Element ICE*Hs*1 found only among our contemporary isolates. Because Cu and Zn are routinely used in feedlot rations, selection pressure created by these metals in *H. somni* might co-select for antimicrobial resistance in *H. somni*.

We identified the *tet*(H) and *ebrB* genes co-located with *mco* and *czcD* genes in the ICE. The *mco* gene encodes a multicopper oxidase enzyme that catalyzes Cu detoxification by converting cuprous ion [Cu(I)] into the less toxic cupric ion [Cu(II)] (44, 45). The cuprous ion can generate reactive oxygen species (ROS) near the cytoplasmic membrane and inner membrane in bacteria that perturb vital cellular process (46). The *czcD* gene encodes a CzcD transporter protein of the cation diffusion facilitator (CDF) family that export divalent metal ions via proton motive force (15, 46). The *acr3* gene, another metal tolerance gene identified in ICE*Hs1*, encodes an arsenical efflux protein (Acr3) which pumps arsenite out of the cell. The *acr3* gene was initially characterized in yeast, but has also been identified in bacteria isolated from poultry farms, including *Campylobacter jejuni* (47).

Genomic comparison of *H. somni* AVI 1 (ICE*Hs*1) with those present in the genomes of *P. multocida* 36950 (ICE*Pmu1*), *M. haemolytica* M42548 (ICE*Mh1*), indicated that ICE*Hs1* is integrated into the fourth of the four copies of tRNA^Leu^, whereas, ICE*Pmu1* and ICE*Mh1* were integrated in the second copy of tRNA^Leu^ (19, 21). ICE*Hs*1 is flanked by the characteristic 11 bp (GATTTGAATC) direct repeats as described earlier in *H. somni* ICE*Hso*2336 and *M. haemolytica* ICE*Mh1* (19, 21, 22). Comparative genomics of these ICEs indicates that ICE*Hs1* shares high sequence identity with ICE*Hso*2336, and the ICEs of BRD pathogens *P. multocida* and *M. haemolytica* (Figure 3). The length of ICE*Hs1* is 64932 bp and it contains 83 genes, as compared to ICE*Mh1* length of 92345 bp with 107 genes, and ICE*Pmu1* length of 82214 bp with 88 genes.

The ICEs in all three BRD pathogens show a mosaic structure with characteristic conserved and variable regions. The conserved region of ICEHs1 shares more than 99% homology with H. somni ICEHso2336, ICEMh1, and ICEPmu1. Among ICE, the variable region has more tolerance for gene insertion and deletion, and contains accessory genes such as those for antimicrobial resistance and metal tolerance. Functions of the open reading frames in ICEHs1 were predicted by sequence comparison using BLASTp. Analysis of coding sequences (CDS) within the conserved region of ICE*Hs*1 identified core genes involved in ICE integration and excision, regulation and maintenance, replication, and conjugative transfer (Figure 3). Among the 83 genes present in ICE*Hs1*, we identified two integrases, three transposases, three metal tolerance genes, two antimicrobial resistance genes, one aldo-keto reductase, two alcohol dehydrogenase gene, 21 genes involved in conjugative transfer, four genes involved in replication or DNA repair, four genes involved in regulation or maintenance of the ICE, and 37 open reading frames with unknown function (Figure 4). It is surprising to note that the relaxase gene, which has been reported in a majority of ICEs was not identified within the sequence ICE*Hs1*, as it is required for self-transmission. It is possible that it is located outside of the ICE sequence, as was seen with the previously described *H. somni* ICE*Hso2336* (22).

The *P. multocida* 36950 strain ICE*Pmu1*, and the *M. haemolytica* 42548 strain ICE*Mh1* each have two variable regions containing antimicrobial resistance genes (Figure 4) (19, 21). The *H. somni* AVI1 strain ICE*Hs1* contains only one variable region which carries tetracycline resistance gene *tet*(H), a multidrug resistance gene (*ebrB*) of the SMR family, the multi-copper oxidase *mco*, a metal binding protein gene, and the *czcD* and *acr3* metal tolerance genes. Comparative genomics shows that *mco* and the metal binding protein gene share 100% sequence identity between ICE*Pmu1*, ICE*Mh1*, and ICE*Hs1*. Of interest, the *czcD* and *acr3* genes present in ICE*Hs1* were absent in ICE*Pmu1* and ICE*Mh1*. An interesting difference between the two *H. somni* ICE's, ICE*Hs1* and ICE*Hso*2336 was the gain of two metal tolerance genes, HEP, and *mco* type 3 in ICE*Hs1*.

The ICE's ICE*Pmu1*, ICE*Mh1*, ICE*Hso*2336, and ICE*Hs1* were all identified in pathogenic field strains isolated from various parts of the USA and Canada at different times, still they are very closely related. The *M. haemolytica* strain 42548, *P. multocida* strain 36950, and *H. somni* strain 2336 were collected respectively from the US states of Pennsylvania, Nebraska, and Washington in 2007, 2005, and 1980's (22). The *H. somni* strain AVI1 from which we first identified ICE*Hs1* was collected in Alberta, Canada in 2012. It is possible that all these elements have a common evolutionary origin, and that regular use of antimicrobials, copper, and zinc in feedlots support their persistence and dispersal.

This study reports antimicrobial resistance profiles of clinical isolates of *H. somni* from feedlot cattle in Alberta, which may be useful information for veterinarians and feedlot managers making treatment and management decisions. The high percentage of oxytetracycline resistance observed in contemporary isolates indicates that including oxytetracycline or chlortetracycline in feedlot rations for prevention and control of BRD and histophilosis may no longer be an effective use of these antimicrobials. The finding of little or no resistance to tilmicosin, tulathromycin, enrofloxacin, ceftiofur, and florfenicol among *H. somni* examined in this study is encouraging, as these important antimicrobials need to remain effective for prevention, control, and treatment of disease in feedlot cattle.

## Author contributions

KL is responsible for the concept and design of the research project, grant funding, and supervision of research personnel. KL contributed to, and edited the manuscript. She is accountable for the accuracy and integrity of the work. KB conducted the laboratory research and statistical analysis, and wrote the majority of the manuscript. ET provided bacterial strains and bovine necropsy tissue for bacterial isolation, and provided guidance to the graduate student. AP contributed to the concept and design, and provided bacterial strains and whole genome sequences for analysis. NR performed sequence analysis, and assisted with creation of figures for the manuscript.

### Conflict of interest statement

The authors declare that the research was conducted in the absence of any commercial or financial relationships that could be construed as a potential conflict of interest.
